# Influences of Postural Control on Cognitive Control in Task Switching

**DOI:** 10.3389/fpsyg.2018.01153

**Published:** 2018-10-05

**Authors:** Denise N. Stephan, Sandra Hensen, Edina Fintor, Ralf Krampe, Iring Koch

**Affiliations:** ^1^Institute of Psychology, RWTH Aachen University, Aachen, Germany; ^2^Brain and Cognition Group, KU Leuven, Leuven, Belgium

**Keywords:** postural control, cognitive control, task switching, task preparation, congruency effect

## Abstract

The aim of the current study was to investigate the effects of postural control demands on cognitive control processes in concurrent auditory-manual task switching. To this end, two experiments were conducted using an auditory cued task-switching paradigm with different postural control demands (sitting vs. standing). This design allowed us to explore the effect of postural control on switch costs, mixing costs, and the between-task congruency effect. In addition, we varied the cue-based task preparation in Experiment 1 to examine whether preparation processes are independent of additional postural control demands or if the motor control processes required by the postural control demands interfere with task-specific cognitive preparation processes. The results show that we replicated the standard effects in task switching, such as switch costs, mixing costs, and congruency effects in both experiments as well as a preparation-based reduction of these costs in Experiment 1. Importantly, we demonstrated a selective effect of postural control demands in task switching in terms of an increased congruency effect when standing as compared to sitting. This finding suggests that particularly in situations that require keeping two tasks active in parallel, the postural control demands have an influence on the degree to which cognitive control enforces a more serial (shielded) mode or a somewhat less selective attention mode that allows for more parallel processing of concurrently held active task rules.

## Introduction

Postural control is crucial in daily life, we depend on it despite the fact that it seems to happen rather effortlessly and automatically. However, studies show significant attentional requirements related to postural control (for a review see Woollacott and Shumway-Cook, 2002) as it refers to the control over a body’s position in space for the purpose of balance and orientation (Woollacott and Shumway-Cook, 2002) and requires the dynamic integration of visual, proprioceptive, and vestibular sensory information (Huxhold et al., 2006). Central aspects of postural control research are the influence of individual preconditions such as age (Donker et al., 2007) or proficiency in balance-related skills and abilities (Krampe et al., 2014), and attentional requirements (Woollacott and Shumway-Cook, 2002). Even though postural control seems to be automatic and effortless, it has been shown that even sitting requires a certain amount of postural motor control (Kerr et al., 1985). It has long been presumed that cognition and motor functions share and thus compete for limited attentional resources (Woollacott, 2000).

Attention can be defined as the information processing capacity of an individual, which is presumably limited (see e.g., Kahneman, 1973; Wickens, 1980, 1989). Usually, studies in the context of postural control used so called cognitive-motor dual-tasks (e.g., Dault et al., 2003) to determine the attentional demand. In these cognitive-motor dual-tasks a postural task (e.g., balancing on a balance board, standing, or walking) and a secondary cognitive task (e.g., counting backward; see Yardley et al., 1999) are performed at the same time and performance is compared to performing only one task separately. According to the notion of limited attentional resources, a more demanding postural task should induce more interference with a cognitive tasks and vice versa (Woollacott and Shumway-Cook, 2002; Fraizer and Mitra, 2008; Boisgontier et al., 2013). However, empirical evidence is ambiguous, as some studies report interference between a motor task and a cognitive task (e.g., Andersson et al., 1998), whereas others did not report an effect of postural control demands (whether participants were sitting or standing) on the performance in the cognitive tasks in general (Dault et al., 2001; see also Huxhold et al., 2006). Other studies tackled this issue but the majority focused on the question whether postural control suffers in terms of for example postural sway and sway velocity increases in cognitive-motor dual tasks compared to single tasks (in this case if a cognitive task is added vs. only a postural task is given; see e.g., Beurskens et al., 2016).

An alternative approach that we took in the present study is to investigate the effects of postural control demands on cognitive processing by using a paradigm, which provides a variety of more specific measures of cognitive control and cognitive flexibility. The task-switching paradigm has long been used as a tool to investigate cognitive control (see Koch et al., 2018). In a typical task-switching paradigm, participants have to perform two or more cognitive tasks (e.g., a parity and a magnitude task) in a certain order and either switch from one task to another (i.e., task switch), or repeat the same task (i.e., task repetition). Usually, performance [i.e., response time (RT) and error rate (ER)] is worse in switch trials relative to repetition trials (Monsell, 2003; Kiesel et al., 2010; Vandierendonck et al., 2010, for reviews). This performance decrement was termed switch costs and is considered a marker of transient, trial-to-trial cognitive control processes dedicated to task switching (Grange and Houghton, 2014). Few studies have used task-set switching in multi-tasking paradigms (for exceptions see Brown et al., 2013; Meijer and Krampe, 2018) and whether or not certain switch specific processes are affected by postural control demands has not been explored yet.

Preparation-based reductions of switch costs have been demonstrated in many studies (e.g., Koch, 2001; Monsell, 2003; Kiesel and Hoffmann, 2004; for reviews see Kiesel et al., 2010; Vandierendonck et al., 2010). With regard to the present study, it was of particular interest whether preparation time could be used independently of additional postural control demands or if the cognitive resources necessary to prepare for the upcoming task interfered with the resources occupied by the postural control demands.

Besides switch costs, mixing costs can be assessed by including single-task blocks in the experimental design as a contrast between repetition trial of the mixed-tasks blocks and performance in single task trials (see e.g., Meiran et al., 2000). Usually RTs are higher and ERs are increased in repetition trials in the mixed-tasks blocks compared to in trials in the single-task blocks (see e.g., Rubin and Meiran, 2005). These so-called mixing costs can be interpreted in terms of higher working memory load, due to the effort of updating and maintaining more than one task set (Kiesel et al., 2010), which refers to the cognitive representation of the task requirements (see Monsell, 2003). The maintenance of the task sets is a necessary precondition for parallel processing of both tasks in mixed task blocks and provides basis for crosstalk between both tasks (see e.g., Fischer and Plessow, 2015). However, a previous study found no effect of postural control demands on working memory tasks (Dault et al., 2001), so that it seems important to include mixing costs as a measure of task set maintenance in the current study to investigate the possible influence of postural control on parallel processing.

The task-switching paradigm does not only allow us to study the influence of postural control demands on cognitive processing with regard to cognitive flexibility (i.e., switch costs) and maintenance of concurrent task sets (i.e., mixing costs), but additionally provides the possibility to determine the influence of postural control demands on between-task interference, measured as the between-task congruency effect (Meiran, 2005). In order for congruency effects to occur, bivalent stimuli that can be applied to either task are necessary. If a stimulus requires the same response for both tasks (e.g., a right keypress), it is congruent, while if it requires different responses (e.g., a right keypress in one and a left keypress in the other task), it is incongruent. The congruency effect denotes the finding that usually participants respond faster to congruent compared to incongruent stimuli (Koch and Allport, 2006). Several authors (e.g., Rosenbaum et al., 1986) have suggested a key role of motor programming in accounting for congruency effects. Accordingly, performance benefits in congruent compared to incongruent trials are due to the maintenance of the appropriate motor programming parameter in memory from trial to trial (e.g., if the same finger is used to respond), while motor parameters must be re-programmed in incongruent trials thereby increasing reaction times. Please note, that we take a broader perspective by considering parameters specifying motor programs as part of the task. In order to explain the congruency effect, it has been argued that incongruent stimuli activate both the response according to the currently relevant task rules (i.e., the relevant task set) and the response according to the currently irrelevant task rules (i.e., the irrelevant task set) of the competing task (Kiesel et al., 2010). The congruency effect reflects the inability to shield the currently relevant task set from the currently irrelevant task set (see e.g., Dreisbach and Haider, 2009; Dreisbach and Wenke, 2011). Thus, while efficient task-set shielding should keep both task-sets distinct and prevent interference when alternating between tasks (i.e., decrease the congruency effect), less efficient task-set shielding would cause parallel processing and thus increase competition between tasks and responses arising in incongruent trails. To our knowledge, no study examined task-set shielding, as measured with the congruency effect in the context of postural control.

In sum, our goal was to determine the influence of postural control demands (sitting vs. standing) on switch costs, mixing costs, congruency effect (as a measure of task-set shielding), and the effects of preparation time before switches. To this end, we used a cued task-switching paradigm, in which cues indicated the currently relevant task and in which preparation time can be manipulated by varying the interval between the cue and the stimulus (CSI). Note that in the task-switching literature, the term single task describes the condition in which only one of the two cognitive tasks is performed (in contrast to a mixed condition in which participants alter between both tasks). Thus different from the cognitive-motor dual-task paradigms described earlier, the single task condition in our approach already constitutes a cognitive-motor dual-task, since a cognitive task is performed either in a condition with low (i.e., sitting) or high (i.e., standing) postural control demand.

Our predictions were based on the assumption that coordinating a cognitive task with a sensorimotor task, even a seemingly automatic one like postural control, taxes cognitive control processes and that this interference is pronounced if postural control demands increase. A key feature of our approach is that the switch condition in the task-switching blocks by itself involves several cognitive control operations, which should be most sensitive to interference from a concurrent postural control task, notably the maintenance and change of task sets and shielding operations to prevent between-task crosstalk. Consequentially, we predicted higher switch costs and stronger congruency effects due to reduced shielding in the standing compared with the sitting condition. Finally, given that task preparation has been shown to reduce though not necessarily eliminate switch costs, we assume that all effects of postural control on task-switching performance should be smaller with long preparation interval.

## Experiment 1

### Methods

#### Participants

Thirty-two participants (25 women; mean age = 22.9 years) took part in the experiment. They all had normal or corrected-to-normal hearing acuity, no balance problems and gave informed consent for participation. Information about their sportiveness was collected after the experiment. There were 5 non-sportive and 27 sportive participants (*M* = 4 h exercise per week since 41 months).

#### Stimuli, Tasks, and Procedure

In the experiment, participants switched between performing an auditory parity (odd or even) and a magnitude task (smaller or greater than five) while sitting or standing. The spoken number words from one to nine (except five) were presented in German binaurally via headphones (Sennheiser PMX 60; words were recorded in cooperation with the Institute of Technical Acoustics at RWTH Aachen University). In both postural control conditions (sit and stand) participants had to look at a visually presented fixation cross. In the sit condition, it was presented at the center of a 17″ screen (6.5 cm × 6.5 cm) with a viewing distance of approximately 78 cm. In the stand condition, the fixation cross was presented on a white wall (15.6 cm × 15.6 cm) with a viewing distance of 143 cm, while participants stand on a foam mat (1 cm height). Postural control demand was manipulated within participants, and the condition order was counterbalanced across participants. Prior to the experiment, participants were asked to take off their shoes. Furthermore, they were asked to complete a questionnaire before and after the experiment.

The experiment was programmed and presented using SR Research Experiment Builder (SR Research Ltd., Mississauga, ON, Canada). Each trial started with an auditory task cue, which was presented for 200 ms, indicating the relevant task (i.e., a 600 Hz sound cued the parity task; 300 Hz sound cued the magnitude task). The duration of the CSI varied randomly from trial to trial (100 ms vs. 1000 ms). In order to keep the response–stimulus interval (RSI) constant at 1100 ms the response-cue interval (RCI) was 1000 ms in trials with a with short CSI (100 ms) and 100 ms in trials with a long CSI (1000 ms). The number words were presented for 470 ms. The magnitude task asked for a smaller or larger than five decision and the parity task for an odd or even judgment. The response was given via left click for even and greater than five and via a right click for odd or smaller than five on the mouse buttons by using either the left or the right thumb. In both, the sitting and the standing condition, participants held the mouse in both hands in front of the upper body. Please note that we did not counterbalance the stimulus–response (S–R) mappings in both tasks but used the less S–R compatible mappings throughout (defined with respect to the SNARC and MARC effect)^1^.

**FIGURE 1:**
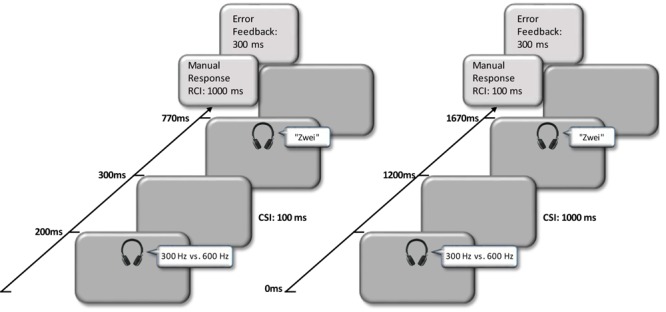
Exemplary overview of trials from the mixed-tasks blocks with either a CSI of 100 ms **(Left)** or a CSI with 1000 ms **(Right)**.

In case of an error, there was an auditory feedback (a twisted 330 Hz sound, created with “Audacity”) was presented for 300 ms, delaying the onset of the next cue. The next trial started after a response was made (for an exemplary overview of individual trials, see **Figure 1**).

**FIGURE 2:**
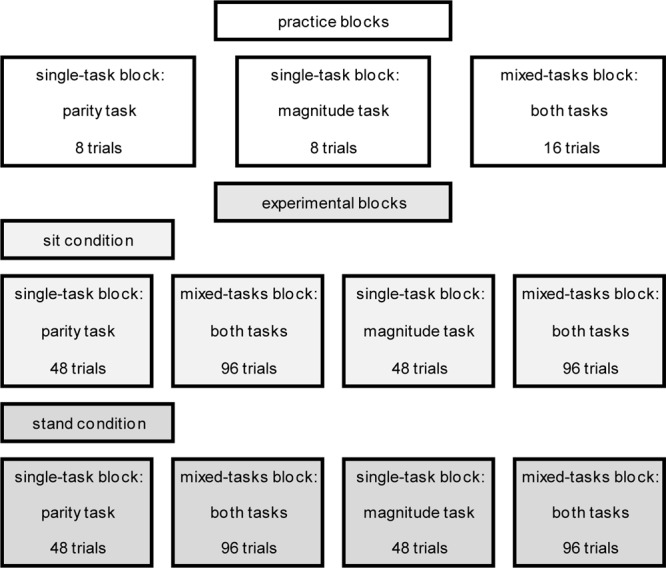
Exemplary overview of the practice and experimental blocks in the sit and stand condition (the order was counterbalanced).

There were three practice blocks [two single-task blocks (parity and magnitude) à eight trials; one mixed-tasks block à 16 trials] at the beginning, which participants performed while sitting, followed by four experimental blocks in each condition [two single-task blocks (parity and magnitude) with 48 trials; two mixed-tasks blocks with 96 trials]. The task order in the mixed-tasks blocks was randomized for each participant individually (i.e., resulting in 50% task repetition trials and 50% task switch trials). Overall, there were eight experimental blocks (for an exemplary overview see **Figure 2**). The order of experimental blocks as well as the postural control demand was counterbalanced across participants. The practice and experimental blocks were separated by short breaks; the start of each block was initiated via mouse click by the participants. Prior to each block, an instruction containing information about the tasks and the S–R mapping was presented. The experiment lasted about 35 to 40 min.

#### Design

The independent within-subject variables were postural control (sit vs. stand), congruence (congruent vs. incongruent), CSI (100 ms vs. 1000 ms), transition (switch vs. repetition in the mixed-task blocks), and mixing (single-task blocks vs. repetition in mixed-tasks blocks). The levels of the variables congruence, CSI, and transition varied randomly, whereas the levels of the variables postural control and mixing were blocked. Single-task performance for the parity task and the magnitude task was analyzed separately. Specifically, we analyzed switch costs (switch trials vs. repetition trials in mixed-tasks blocks) and mixing costs (single-task tasks vs. repetition trials in mixed-tasks blocks) separately as two non-orthogonal contrasts. The dependent variables were RT and ER. All tests of significance were conducted at an alpha level of 0.05.

### Results

For data analysis, all practice blocks and the first two trials of each block were removed to account for restart costs (cf. Allport and Wylie, 2000). Moreover, all trials exceeding a *z*-score of -3/+3 (*z*-transformation of all RTs for each participant separately) were discarded as outliers (1.9%). Additionally, for the RT analysis, we excluded all erroneous trials (7.7%), as well as trials after an error. For an overview of the significant results please see **Appendixes A1, A2**.

#### Mixing Costs Analysis

A repeated measure ANOVA with the independent variables postural control (sit vs. stand), congruence (congruent vs. incongruent), mixing (single-task blocks vs. repetition in mixed-tasks blocks), and CSI (100 ms vs. 1000 ms; mean RTs and ERs are presented in **Table 1**) was conducted. For RT, it revealed a significant main effect of postural control [*F*(1,31) = 4.39; *p* < 0.05; $\eta_{p}^{2}$ = 0.124], surprisingly, indicating higher RTs in the sit condition (827 ms) than in the stand condition (788 ms). The main effect of congruence was significant, too [*F*(1,31) = 42.81; *p* < 0.001; $\eta_{p}^{2}$ = 0.580], indicating higher RTs in incongruent trials (843 ms) compared to congruent trials (772 ms). Furthermore, we found significant mixing costs [*F*(1,31) = 55.40; *p* < 0.001; $\eta_{p}^{2}$ = 0.641], indicating higher RTs in repetition trials of mixed-tasks blocks (921 ms) than in single-task blocks (693 ms). Also the main effect of CSI was significant [*F*(1,31) = 23.21; *p* < 0.001; $\eta_{p}^{2}$ = 0.428], indicating higher RTs in trials with a CSI of 100 ms (831 ms) compared to trials with a CSI of 1000 ms (784 ms).

**Table 1:** RT (ms) and ER (%) (SD in parentheses) data of Experiment 1 for single, repetition, and switch trials as a function of postural control (sit vs. stand), congruence (congruent vs. incongruent and congruence effect), and CSI (100 ms vs. 1000 ms).

		Congruent		Incongruent		Congruence effect		Congruent		Incongruent		Congruence effect	
Condition		100	1000	100	1000	100	1000	100	1000	100	1000	100	1000

Single	Sit	700 (146)	699 (139)	723 (169)	711 (139)	23	12	3.5 (4.6)	4.2 (5.6)	4.9 (5.7)	3.6 (3.5)	1.4	–0.6
	Stand	660 (106)	660 (114)	700 (127)	691 (111)	40	31	3.4 (5.8)	3.0 (4.3)	4.8 (5.0)	6.1 (6.1)	1.4	3.1
Repetition	Sit	945 (300)	848 (223)	1040 (364)	949 (307)	95	101	2.8 (5.1)	2.3 (4.2)	10.4 (8.6)	9.0 (10.2)	7.6	6.7
	Stand	862 (217)	798 (230)	1016 (341)	913 (240)	154	115	3.6 (5.3)	1.9 (3.6)	10.6 (7.6)	10.5 (9.1)	7	8.6
Switch	Sit	1073 (297)	931 (313)	1198 (414)	1005 (323)	125	74	3.9 (5.2)	4.0 (5.3)	16.7 (13.5)	18.3 (13.9)	12.8	14.3
	Stand	1014 (249)	865 (215)	1153 (285)	972 (266)	139	107	5.3 (6.3)	3.0 (4.9)	18.3 (11.6)	16.0 (12.5)	13	13

**FIGURE 3:**
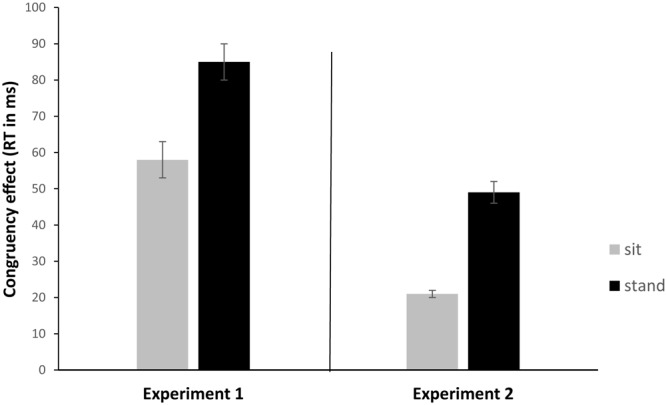
Congruency effect (RT in ms) in Experiment 1 averages across CSIs and Experiment 2 (mixing cost analysis) as a function of postural control (sit vs. stand). Error bars indicate standard deviation.

Most importantly in the present context, the congruency effect tended to be larger when standing compared to sitting (85 ms vs. 58 ms), as suggested by a non-significant trend for the interaction of postural control and congruence [*F*(1,31) = 3.42; *p* = 0.074; $\eta_{p}^{2}$ = 0.099; see **Figure 3**]. Please note that this interaction (i.e., the increased congruency effect in task switching in the standing condition) was significant in Experiment 2. The interaction between congruence and mixing was significant [*F*(1,31) = 17.29; *p* < 0.001; $\eta_{p}^{2}$ = 0.358], indicating a larger congruency effect in repetition trials in mixed-tasks blocks compared to single-task blocks (116 ms vs. 26 ms). Also the interaction between CSI and mixing was significant [*F*(1,31) = 16.30; *p* < 0.001; $\eta_{p}^{2}$ = 0.345], indicating a larger benefit of preparation in repetition trials in mixed-tasks blocks compared to single-task blocks (89 ms vs. 6 ms). All other interactions were not significant (*F* < 1).

The same ANOVA on ERs (mean RTs and ERs are presented in **Table 1**) showed a significant main effect of congruence [*F*(1,31) = 42.73; *p* < 0.001; $\eta_{p}^{2}$ = 0.580], indicating increased ERs in incongruent trials (7.5%) compared to congruent trials (3.1%). Furthermore, we found significant mixing costs, indicating increased ERs in repetition trials in mixed-tasks blocks (6.4%) than in single-task blocks (4.2%). The main effect of postural control and CSI was not significant (*F* < 1).

Also the interaction between congruence and mixing was significant [*F*(1,31) = 29.09; *p* < 0.001; $\eta_{p}^{2}$ = 0.484], indicating a larger congruency effect in repetition trials in mixed-tasks blocks compared to single-task blocks (7.5% vs. 1.4%). There was also a non-significant trend toward an interaction between postural control, congruence and CSI [*F*(1,31) = 3.10; *p* = 0.088; $\eta_{p}^{2}$ = 0.091], hence numerically the influence of postural control on congruency was larger with a long CSI (congruency effect: 3.1% sitting vs. 5.9% standing) compared to shorter CSI (4.5% sitting vs. 4.2% standing). All other interactions were not significant; for postural control and congruence [*F*(1,31) = 1.59; *p* = 0.216; $\eta_{p}^{2}$ = 0.049], for mixing and CSI [*F*(1,31) = 1.18; *p* = 0.286; $\eta_{p}^{2}$ = 0.037]; for all other interactions (*F* < 1).

#### Task-Switching Analysis

A repeated measures ANOVA with the independent variables postural control (sit vs. stand), congruence (congruent vs. incongruent), task transition (switch vs. repetition), and CSI (100 ms vs. 1000 ms) was conducted only using performance in mixed-tasks blocks (mean RTs and ERs are presented in **Table 2**). For RT, it revealed a significant main effect of congruence, indicating longer RTs in incongruent trials (1031 ms) compared to congruent trials [917 ms; *F*(1,31) = 34.81; *p* < 0.001; $\eta_{p}^{2}$ = 0.529]. The main effect of transition was significant, too, indicating longer RTs in switch trials (1026 ms) compared to repetition trials [921 ms; *F*(1,31) = 50.28; *p* < 0.001; $\eta_{p}^{2}$ = 0.619]. Furthermore, we found a significant main effect of CSI, RTs were significantly longer in trials with a CSI of 100 ms (1038 ms) than in trials with a CSI of 1000 ms [910 ms; *F*(1,31) = 80.94; *p* < 0.001; $\eta_{p}^{2}$ = 0.723]. The main effect of postural control was not significant [*F*(1,31) = 2.29; *p* = 0.141; $\eta_{p}^{2}$ = 0.069].

**Table 2:** RT (ms) and ER (%) (SD in parentheses) data of Experiment 2 for single, repetition, and switch trials as a function of postural control (sit vs. stand) and congruence (congruent vs. incongruent and congruence effect).

Condition		Congruent	Incongruent	Congruence effect	Congruent	Incongruent	Congruence effect
Single	Sit	688 (111)	701 (114)	13	4.1 (4.3)	4.1 (3.9)	0
	Stand	674 (109)	698 (120)	24	3.9 (6.0)	3.5 (3.8)	–0.4
Repetition	Sit	939 (245)	969 (244)	30	3.1 (4.3)	7.7 (6.7)	4.6
	Stand	926 (236)	1000 (255)	74	3.7 (4.1)	7.4 (5.8)	3.7
Switch	Sit	1124 (298)	1177 (305)	53	5.2 (5.9)	12.1 (6.9)	6.9
	Stand	1098 (306)	1180 (380)	82	4.9 (6.2)	15.8 (11.9)	10.9

Furthermore, the interaction between transition and CSI was significant [*F*(1,31) = 19.41; *p* < 0.001; $\eta_{p}^{2}$ = 0.385], indicating higher switch costs in trials with a CSI of 100 ms than in trials with a CSI of 1000 ms (167 ms vs. 89 ms). No other interactions were significant; for postural control and congruence [*F*(1,31) = 1.45; *p* = 0.231; $\eta_{p}^{2}$ = 0.046]; for congruence and CSI [*F*(1,31) = 1.76; *p* = 0.195; $\eta_{p}^{2}$ = 0.054]; for the four-way interaction between postural control, congruence, CSI, and transition [*F*(1,31) = 1.12; *p* = 0.301; $\eta_{p}^{2}$ = 0.034], for all other interactions (*F* < 1).

The same ANOVA on ERs (mean RTs and ERs are presented in **Table 2**) showed a significant main effect of transition [*F*(1,31) = 40.92; *p* < 0.001; $\eta_{p}^{2}$ = 0.569], indicating that ERs were higher on switch trials (10.7%) than on repetition trials (6.4%). The main effect of congruence was significant, too [*F*(1,31) = 74.22; *p* < 0.001; $\eta_{p}^{2}$ = 0.705], indicating higher ERs in incongruent trials (13.7%) than in congruent trials (3.4%). Neither the main effect of postural control (*F* < 1), nor the main effect of CSI [*F*(1,31) = 1.85; *p* = 0.184; $\eta_{p}^{2}$ = 0.056] was significant. There was a significant interaction between congruence and transition [*F*(1,31) = 17.28; *p* < 0.001; $\eta_{p}^{2}$ = 0.358], indicating larger switch costs in incongruent trials than in congruent trials (7.2% vs. 1.5%). There was also a non-significant trend toward an interaction between postural control, transition, and CSI [*F*(1,31) = 3.30; *p* = 0.079; $\eta_{p}^{2}$ = 0.096], hence numerically with a short CSI switch costs were smaller when sitting compared to standing (switch costs: 3.7% sitting vs. 4.7% standing) the pattern was reversed with a long CSI (switch costs: 5.6% sitting vs. 3.3% standing). All other interactions were non-significant; for postural control and CSI [*F*(1,31) = 2.78; *p* = 0.105; $\eta_{p}^{2}$ = 0.082]; for postural control, congruence, transition and CSI [*F*(1,31) = 1.54; *p* = 0.225; $\eta_{p}^{2}$ = 0.047]; for all other interactions (*F* < 1).

### Discussion

In Experiment 1, we found significant switch costs, mixing costs, and a congruency effect in RTs and ER. Furthermore, an effect of preparation was present in terms of shorter RTs on trials with a long CSI compared to trials with a short CSI as well as reduced switch and mixing costs on trials with a long CSI compared to trials with short CSI. With regard to the influence of postural control demands, there was a non-significant numerical trend depicting faster responses when standing compared to sitting. However, there was no other interaction between CSI and postural control, so it does not seem that the benefit of preparation is affected by postural control demands. Further, neither the interaction between postural control and task transition nor between postural control and mixing was significant, thus the postural control demand does not seem to directly affect switch costs or mixing costs.

Importantly, even though the interaction between postural control and congruency failed to reach significance by a slight margin, there was a numerical trend regarding a larger congruency effect when standing compared to sitting. In order to follow up this effect, a second experiment was conducted in which we used a constant CSI of medium duration (400 ms).

## Experiment 2

### Methods

#### Participants

Twenty-four participants (21 women; mean age = 23.3 years) took part in the experiment. They all had normal or corrected-to-normal hearing acuity, no balance problems and gave informed consent for participation. They received course credits for participation. Information about the sportiness were collected after the experiment. There were 2 non-sportive and 22 sportive participants (*M* = 5 h exercise per week since 115 months).

#### Stimuli, Tasks, Procedure, and Design

Stimuli, tasks, and procedures in Experiment 2 were identical to Experiment 1, the only difference being, that the CSI was held constant at 400 ms. The independent within-subject variables were postural control (sit vs. stand), congruence (congruent vs. incongruent), transition (switch vs. repetition), and mixing (single-task blocks vs. repetition trials in mixed-tasks blocks). Data analyses proceeded as in Experiment 1.

### Results

All practice blocks and the first two trials of each experimental block were discarded for all analyses. Moreover, we excluded all outliers by performing *z*-transformations of all RTs for each participant separately. Trials with a *z*-score of -3/+3 were discarded as outliers (1.8%). Additionally, for the RT analysis, we excluded all erroneous trials (6.8%), as well as trials following errors. For an overview of significant results please see **Appendixes A3, A4**.

#### Mixing Costs Analysis

A repeated measure ANOVA with the independent variables postural control (sit vs. stand), congruence (congruent vs. incongruent), and mixing (single-task blocks vs. mixed-tasks blocks), only using performance of single-task block and the repetition trials from the mixed-tasks blocks (mean RTs and ERs are presented in **Table 2**) was conducted. As in Experiment 1, it showed a significant main effect congruence, indicating higher RTs in incongruent trials (842 ms) compared to congruent trials {807 ms; [*F*(1,23) = 14.36; *p* = 0.001; $\eta_{p}^{2}$ = 0.384], and of mixing [*F*(1,23) = 64.42; *p* < 0.001; $\eta_{p}^{2}$ = 0.737]}, indicating higher RTs in mixed-tasks blocks (958 ms) than in single-task blocks (690 ms). The main effect of postural control was not significant (*F* < 1).

Most importantly, we found a significant interaction between postural control and congruence [*F*(1,23) = 6.08; *p* < 0.005; $\eta_{p}^{2}$ = 0.209]. This interaction indicates a larger congruency effect when standing (49 ms) compared to sitting (21 ms) and thus replicates the almost significant trend (*p* = 0.074) that we observed in Experiment 1 (85 ms vs. 58 ms; see **Figure 3**).

Also, the interaction between congruence and mixing was significant [*F*(1,23) = 4.73; *p* < 0.005; $\eta_{p}^{2}$ = 0.170], indicating a larger congruency effect in mixed-tasks (51 ms) compared to single-task blocks (18 ms). No other interaction was significant; for postural control, congruence, and mixing [*F*(1,23) = 1.04; *p* = 0.318; $\eta_{p}^{2}$ = 0.043], for all other (*F* < 1).

As in Experiment 1, the same ANOVA on ERs showed a significant main effect of congruence [*F*(1,23) = 10.02; *p* < 0.05; $\eta_{p}^{2}$ = 0.303], indicating increased ERs in incongruent (5.7%) compared to congruent trials (3.7%), of mixing [*F*(1,23) = 12.16; *p* < 0.05; $\eta_{p}^{2}$ = 0.346], indicating increased ERs in mixed-tasks blocks (5.5%) than in single-task block {3.9%, and the interaction [*F*(1,23) = 17.23; *p* < 0.001; $\eta_{p}^{2}$ = 0.428]}, indicating a larger congruency effect in mixed-tasks blocks (4.2%) compared to single-task blocks (-0.2%). No other effect or interaction was significant (*F* < 1).

In sum, in the mixing-costs analysis of Experiment 2, the main effects demonstrated in Experiment 1 were nicely replicated. Importantly, the numerical trend toward an interaction between postural control and congruency in mixing costs could be replicated in RTs, thus providing converging evidence for an influence of postural control demands.

#### Task-Switching Analysis

A repeated measures ANOVA with the independent variables postural control (sit vs. stand), congruence (congruent vs. incongruent) and transition (switch vs. repetition), only using performance of mixed-tasks blocks (mean RTs and ERs are presented in **Table 2**) was conducted. For RT, as in Experiment 1, it showed a significant main effect of congruence, indicating longer RTs in incongruent trials (1081 ms) compared to congruent trials [1022 ms; *F*(1,23) = 9.65; *p* < 0.05; $\eta_{p}^{2}$ = 0.296] and a significant main effect of transition, indicating longer RTs in switch trials (1145 ms) compared to repetition trials [958 ms; *F*(1,23) = 32.09; *p* < 0.001; $\eta_{p}^{2}$ = 0.582]. The main effect of postural control was not significant (*F* < 1).

Like in the mixing-costs analysis, we found a significant interaction between postural control and congruence [*F*(1,23) = 5.37; *p* < 0.05; $\eta_{p}^{2}$ = 0.189], indicating a larger congruency effect when standing (78 ms) compared to sitting (41 ms). Please note that this interaction was not present in Experiment 1. No other interaction was significant (*F* < 1).

The same ANOVA on ERs replicated the main effects and interaction demonstrated in Experiment 1: we found a main effect of congruence [*F*(1,23) = 39.40; *p* < 0.001; $\eta_{p}^{2}$ = 0.631], indicating increased ERs in incongruent trials (10.8%) compared to congruent trials (4.2%), a main effect of transition [*F*(1,23) = 57.63; *p* < 0.001; $\eta_{p}^{2}$ = 0.715], indicating increased ERs in switch (9.5%) compared with repetition trials (5.5%), and an interaction between congruence and transition [*F*(1,23) = 15.82; *p* = 0.001; $\eta_{p}^{2}$ = 0.408], reflecting larger congruency effects in switch (6.4%) compared with repetition trials (1.6%).

The main effect of postural control was not significant (*F* < 1), but the interaction between postural control, congruence, and transition was significant [*F*(1,23) = 7.17; *p* < 0.05; $\eta_{p}^{2}$ = 0.238]. A follow-up two-way ANOVA, conducted separately for repetition and switch trials showed that, while for switch trials, there was a non-significant numerical trend toward an interaction between postural control and congruence [*F*(1,23) = 3.27; *p* = 0.084; $\eta_{p}^{2}$ = 0.124], suggesting a larger congruency effect while sitting (4.6%) compared to standing (3.7%) this trend was not present for the repetition trials (*F* < 1).

## General Discussion

The aim of the current study was to investigate the effects of postural control demands on cognitive control processes in concurrent cognitive task switching. To this end, we combined an auditory cued task-switching paradigm with manual responses with different postural control demands (sitting vs. standing). This design allowed us to explore the effect of postural control on specific component processes of cognitive control, namely switch costs, mixing costs, and the between-task congruency effect. In addition, we were interested to see whether cue-based task preparation processes are independent of additional postural control demands or if the motor control processes required by the postural control demands interfere with task-specific cognitive preparation processes.

We replicated the standard effects in task switching, such as switch costs, mixing costs, and congruency effects in both experiments (for reviews see Kiesel et al., 2010; Vandierendonck et al., 2010; Koch et al., 2018). The main difference between Experiment 1 and Experiment 2 was the manipulation of preparation time (CSI). We demonstrated the expected influence of CSI including preparation-based reduction of switch and mixing costs. At the same time, these effects appeared to be independent of our postural control manipulation. For the remainder of the discussion, we focus on the effects of postural control demands on cognitive control processes in single- and switching tasks.

In both experiments, the effects of a concurrent postural control task on single-task performance (i.e., in pure blocks of the parity task alone or the magnitude task alone) and performance in mixed tasks blocks did not differ between sitting and standing conditions. We did not find an influence of postural control on mixing costs suggesting that increased postural control demands while standing do not interfere with the working-memory maintenance of the task set in task-repetition trials in mixed blocks. This finding is in line with the study of Dault et al. (2001) who found no costs in WM performance. Further, we did not find a significant influence of postural control on specific task-switch costs, suggesting that increasing postural control demands do not lead to additional interference with the processes underlying an instructed switch of tasks over and above what we demonstrated for mixing costs.

Note, however, that this conclusion is limited to performance in the auditory task-switching paradigm, because we did not assess potential costs in postural control. The most prominent effect in our view relates to the effects of increased postural control demands on task set shielding assessed through the congruency effect. In mixed-task blocks, the congruency effect was numerically larger compared to the single task blocks, and congruency was increased while standing as compared to sitting. This effect on the size of the congruency effect differed across experiments and type of analyses slightly, but the overall direction of this influence of postural control was consistent. Note that even though there was a congruency effect in single tasks, it did not differ with postural control demands in this condition.

As described earlier, the congruency effect is a measure for between-task interference (Meiran, 2005). If participants alternate between two tasks, they must keep task sets and rules distinct enough to prevent interference. Several authors have argued for a shielding function that protects from interference and helps focusing attention on the relevant task by increasing selectivity of processing (e.g., Dreisbach and Haider, 2008, 2009). In incongruent trials, the irrelevant stimulus feature can activate a competing response instantiating the currently irrelevant S–R rule, which creates task interference that has to be resolved (Kiesel et al., 2010). Better task set shielding should keep the congruency effect small. Conversely, less efficient task-set shielding would increase the degree of parallel processing and thus the degree of task and response competition that arises on incongruent trials. Our findings suggest that shielding is less efficient when task sets need to be switched in the mixed blocks, in particularly if cognitive control processes are already occupied by a concurrent postural control task. From a slightly different perspective, one might argue that the main difference between single tasks and mixed tasks is that tasks are processed strictly serially in single task blocks and only one task set is necessary to perform the task successfully. In contrast, both task sets need to be kept active in the mixed tasks blocks, so tasks can be processed, to some degree, in parallel (see also Fischer and Plessow, 2015). If this situation is aggravated by increased concurrent postural control demands (i.e., standing relative to sitting), shielding might become more difficult. In the context of the present study, we demonstrated that particularly in this situation, implying a parallel processing of both tasks, there is an influence of postural control demands. At a more general level, this provides evidence that motor control demands influence the degree of task-set shielding and thus demonstrate that motor control and cognitive control in task switching are not independent but interact with each other.

Besides mixing costs, switch costs, as a measure of cognitive flexibility, were assessed (Kiesel et al., 2010; Vandierendonck et al., 2010, for reviews). We found that switch costs were substantial, but that they are not affected by the postural control demand (see also Dault et al., 2001). This suggests that the task switch itself, that is, the encoding of new instruction and the change of the currently relevant task rules refers to a set of processes that are unrelated to motor control in the sense that they function independently of whether participants are generally in a mode that encourages a more serial or more parallel processing mode (i.e., more or less shielded task sets). It is also noteworthy that overall performance level was not affected by postural control demands, suggesting that these demands have a highly specific influence on a subset of cognitive control processes, notably a cognitive control parameter that specifies the degree to which parallel processing is allowed (Woollacott and Shumway-Cook, 2002, for a review). This finding thus adds to a growing number of findings suggesting that the degree of serial vs. parallel processing in multitasking is not structurally determined but can vary with contextual factors (Fischer and Plessow, 2015).

## Conclusion

In conclusion, the present study demonstrated an effect of postural control demands in task switching in terms of an increased congruency effect. It seems that particularly in situations that require keeping two tasks active in parallel, the postural control demands have an influence on the degree to which cognitive control enforces a more serial (shielded) mode or a somewhat less selective attention mode that allows for more parallel processing of concurrently held active task rules. Future work is desirable to explore how exactly the difference of postural control in standing vs. sitting translates into this specific bias to process tasks less serially when standing.

## Ethics Statement

This study was carried out in accordance with the ethical guidelines of the German Psychological Society (Deutsche Gesellschaft für Psychologie) with written informed consent from all subjects. Ethical review and approval was not required for this study in accordance with the national and institutional guidelines. All subjects gave written informed consent in accordance with the Declaration of Helsinki.

## Author Contributions

DS developed the idea together with IK and frequently discussed it with RK, SH, and EF. DS wrote the manuscript, while IK, RK, SH, and EF contributed by giving feedback to improve the manuscript. SH also tested the participants of Experiment 1 and programmed the experiments.

## Conflict of Interest Statement

The authors declare that the research was conducted in the absence of any commercial or financial relationships that could be construed as a potential conflict of interest.

## Footnotes

1 The SNARC (spatial-numerical association of response codes) effect implies that participants associate small numbers with the left and large numbers with right on a mental number line (Dehaene et al., 1993). The MARC (linguistic markedness of response codes) effect implies that responses are facilitated if stimuli and response codes both have the same (congruent) linguistic markedness (even-right; uneven-left; e.g., Nuerk et al., 2004).

## Appendix

**Table A1:** An overview of the significant results of the mixing costs analyses in Experiment 1.

		Experiment 1
		**Mixing costs analysis**

Postural control	RT	*F*(1,31) = 4.39; *p* < 0.05; $\eta_{p}^{2}$ = 0.124
Congruence	RT ER	*F*(1,31) = 42.81; *p* < 0.001; $\eta_{p}^{2}$ = 0.580 *F*(1,31) = 42.73; *p* < 0.001; $\eta_{p}^{2}$ = 0.580
Mixing	RT	*F*(1,31) = 55.40; *p* < 0.001; $\eta_{p}^{2}$ = 0.641
	ER	*F*(1,31) = 14.88; *p* = 0.001; $\eta_{p}^{2}$ = 0.324
CSI	RT	*F*(1,31) = 23.21; *p* < 0.001; $\eta_{p}^{2}$ = 0.428
Postural control × congruence	RT	*F*(1,31) = 3.42; *p* = 0.074; $\eta_{p}^{2}$ = 0.099
Congruence × mixing	RT ER	F(1,31) = 17.29; *p* < 0.001; $\eta_{p}^{2}$ = 0.358 *F*(1,31) = 29.09; *p* < 0.001; $\eta_{p}^{2}$ = 0.484
CSI × mixing	RT	*F*(1,31) = 16.30; *p* < 0.001; $\eta_{p}^{2}$ = 0.345

**Table A2:** An overview of the significant results of the task-switching analyses in Experiment 1.

		Experiment 1
		**Task-switching analysis**

Congruence	RT ER	*F*(1,31) = 34.81; *p* < 0.001; $\eta_{p}^{2}$ = 0.529 *F*(1,31) = 74.22; *p* < 0.001; $\eta_{p}^{2}$ = 0.705
Transition	RT	*F*(1,31) = 50.28; *p* < 0.001; $\eta_{p}^{2}$ = 0.619
	ER	*F*(1,31) = 40.92; *p* < 0.001; $\eta_{p}^{2}$ = 0.569
CSI	RT	*F*(1,31) = 80.94; *p* < 0.001; $\eta_{p}^{2}$ = 0.723
Transition × CSI	RT	*F*(1,31) = 19.41; *p* < 0.001; $\eta_{p}^{2}$ = 0.385
Congruence × transition	ER	*F*(1,31) = 17.28; *p* < 0.001; $\eta_{p}^{2}$ = 0.358

**Table A3:** An overview of the significant results of the mixing costs analyses in Experiment 2.

		Experiment 2
		**Mixing costs analysis**

Congruence	RT ER	*F*(1,23) = 14.36; *p* = 0.001; $\eta_{p}^{2}$ = 0.384 *F*(1,23) = 10.02; *p* < 0.05; $\eta_{p}^{2}$ = 0.303
Mixing	RT	*F*(1,23) = 64.42; *p* < 0.001; $\eta_{p}^{2}$ = 0.737
	ER	*F*(1,23) = 12.16; *p* < 0.05; $\eta_{p}^{2}$ = 0.346
Postural control × congruence	RT	*F*(1,23) = 6.08; *p* < 0.005; $\eta_{p}^{2}$ = 0.209
Congruence × mixing	RT ER	*F*(1,23) = 4.73; *p* < 0.005; $\eta_{p}^{2}$ = 0.170 *F*(1,23) = 17.23; *p* < 0.001; $\eta_{p}^{2}$ = 0.428

**Table A4:** An overview of the significant results of the task-switching analyses in Experiment 2.

		Experiment 2
		**Task switching analysis**

Congruence	RT ER	*F*(1,23) = 9.65; *p* < 0.05; $\eta_{p}^{2}$ = 0.296 *F*(1,23) = 39.40; *p* < 0.001; $\eta_{p}^{2}$ = 0.631
Transition	RT ER	*F*(1,23) = 32.09; *p* < 0.001; $\eta_{p}^{2}$ = 0.582 *F*(1,23) = 57.63; *p* < 0.001; $\eta_{p}^{2}$ = 0.715
Postural control × congruence	RT	*F*(1,23) = 5.37; *p* < 0.05; $\eta_{p}^{2}$ = 0.189
Congruence × transition	ER	*F*(1,23) = 15.82; *p* = 0.001; $\eta_{p}^{2}$ = 0.408
Postural control × congruence × transition	ER	*F*(1,23) = 7.17; *p* < 0.05; $\eta_{p}^{2}$ = 0.238
